# Avoiding bias due to perfect prediction in multiple imputation of incomplete categorical variables^☆^

**DOI:** 10.1016/j.csda.2010.04.005

**Published:** 2010-10-01

**Authors:** Ian R. White, Rhian Daniel, Patrick Royston

**Affiliations:** a MRC Biostatistics Unit, Institute of Public Health, Cambridge CB2 0SR, Cambridge, UK; b Medical Statistics Unit, London School of Hygiene and Tropical Medicine, London, UK; c Hub for Trials Methodology Research, MRC Clinical Trials Unit and University College London, London, UK

**Keywords:** Missing data, Multiple imputation, Perfect prediction, Separation

## Abstract

Multiple imputation is a popular way to handle missing data. Automated procedures are widely available in standard software. However, such automated procedures may hide many assumptions and possible difficulties from the view of the data analyst. Imputation procedures such as monotone imputation and imputation by chained equations often involve the fitting of a regression model for a categorical outcome. If perfect prediction occurs in such a model, then automated procedures may give severely biased results. This is a problem in some standard software, but it may be avoided by bootstrap methods, penalised regression methods, or a new augmentation procedure.

## 1 Introduction

Multiple imputation (MI) is a popular way to handle missing data under the missing at random assumption (MAR) (Little and Rubin, 2002). Briefly, the missing data are stochastically imputed m times. In the commonest approach, the m completed data sets are then analysed using methods appropriate for complete data, and the m results are combined using Rubin’s rules (Rubin, 1987a).

The main difficulty in MI lies in designing a suitable method to perform the imputations. In particular, Rubin’s rules will only give valid standard errors if the imputations adequately reflect the uncertainty in the data (i.e. they are “proper” (Rubin, 1987a)). Three main methods are available in standard software: a multivariate normal procedure and two procedures based on univariate regressions.

First, a multivariate normal distribution may be assumed for the data, and a Monte Carlo Markov Chain (MCMC) procedure may be used to draw samples of the missing data from their posterior distribution given the observed data. This is implemented in SAS (SAS Institute Inc., 2004) and Stata (StataCorp, 2009), as user-written software in S-PLUS (Schafer, 2008), and in stand-alone software (Schafer, 2008). It is also advocated for data including categorical variables (Schafer, 1997), but a normal distribution for such variables is unlikely to perform well in general (Bernaards et al., 2007).

Second, if the missing data have a monotone pattern, then it is possible to impute the variables in increasing order of amount of missing data, using appropriate generalised linear models of each variable on the previous variables. This method avoids the normality assumption in the first approach and has been implemented in SAS (SAS Institute Inc., 2004) and Stata (StataCorp, 2009).

A third option is multiple imputation by chained equations (MICE) (van Buuren et al., 1999), also known as fully conditional specification (van Buuren, 2007) and sequential regression multivariate imputation (Raghunathan et al., 2001). This starts by filling in missing values in any convenient way and then imputes each variable in turn, using a regression of the observed values of that variable on the observed and currently imputed values of all other variables. As with the monotone method, appropriate generalised linear models (GLMs) are used. This method has been implemented as user-written software in Stata (Royston, 2004, 2005, 2007, 2009), S-PLUS or R (van Buuren and Oudshoorn, 2000) and SAS (Raghunathan et al., 2001, 2007).

In order for imputations to be proper, the imputation procedure must account for uncertainty in the parameters of the imputation model. This is achieved automatically in the MCMC procedure, but the regression-based approaches require a two-step procedure: (1) draw the regression parameters from their posterior distribution, and (2) draw the imputed values from the regression model using the sampled regression parameters. The first step is computationally straightforward in linear regression. With other GLMs, the posterior is commonly approximated by a multivariate normal distribution, from which it is easy to draw parameters: we call this the “Normal-approximation draw” method. This technique will clearly run into difficulties if the Normal approximation is poor.

This paper focuses on a situation where the Normal approximation is very poor: when imputing a discrete variable for which “perfect prediction” (or “separation” (Heinze and Schemper, 2002)) occurs. For example, in logistic regression, perfect prediction occurs if there is a level of a categorical explanatory variable for which the observed values of the outcome are all one (or all zero). In this case, the likelihood increases to a limit as one or more model coefficients go to plus or minus infinity.

The paper explores the implications of perfect prediction for MI using monotone or MICE imputation with the “Normal-approximation draw” method, and proposes several solutions. Section 2 introduces a trial in dental pain with a repeated binary outcome, in which different software packages, apparently implementing the same algorithm, give very different results. Section 3 describes proper imputation and perfect prediction. Section 4 shows possible pitfalls with the aid of a simple artificial data set. Section 5 proposes a way round the problem that may be included in an automated procedure. Section 6 returns to the analysis of the dental pain trial. Section 7 reports a simulation study, and Section 8 discusses broader issues.

## 2 Dental pain data

Our example comes from a clinical trial exploring the treatment of moderate or severe pain in patients who have had their third molar extracted. These data were also analysed by Carpenter and Kenward (2008). 366 patients were randomly assigned to one of seven treatment groups: an active drug A at 5 different doses, a control drug C, or placebo. The outcome to be analysed is a binary measure of pain relief, 1 indicating some or complete relief from pain and 0 indicating no relief, measured 0.25, 0.5, 0.75, 1, 1.5, 2, 3, 4, 5 and 6 h after tooth extraction.

We define $Y_{tij}$ as the outcome at the jth occasion for the ith individual randomised to treatment t, where j=1,…,10 and t=1,…,5 for the 5 doses of drug A, t=6 for drug C and t=7 for placebo. The aim of the present analysis is to compare the prevalence of pain relief at the final time-point across the 7 treatments, so we are interested in contrasts of the $\delta_{t}$ in the simple analysis model

(1) $$\mathrm{logit}\;p\left(Y_{ti10}=1\right)=\delta_{t}\text{.}$$

Although it would be more usual to have a model with an intercept and 6 contrasts, having one parameter per arm makes it easier to explore the impact of perfect prediction.

Unfortunately, only 216 patients had $Y_{ti10}$ observed, because 87, 34, 10, 8 and 11 patients were lost to follow-up after the 5th, 6th, 7th, 8th and 9th observations respectively. Loss to follow-up was strongly associated with previous lack of pain relief, so the missing data are a likely source of bias in a complete cases (CC) analysis, which excludes individuals with missing $Y_{ti10}$. The missing data pattern was very nearly monotone; to simplify analysis, we made it completely monotone by replacing 5 intermittent missing values at the 4th, 5th and 6th observations with the previously observed values. We do not advocate such a procedure in general, but the handling of the intermittent missing values is unimportant in these data: alternative procedures, imputing the 5 intermittent missing values all as 0 or all as 1, produced results differing by less than 1% of a standard error from those shown.

Constructing an imputation model for these data is problematic because outcomes change very little over time: over 40% of patients were never observed to change their outcome, and only 10% changed their response more than once during follow-up. We therefore follow Carpenter and Kenward (2008) and allow each outcome to depend on the previous outcome but not on earlier outcomes, with the associations between outcomes being allowed to vary over time and between treatment groups. This yields the imputation model

(2) $$\mathrm{logit}\;p\left(Y_{tij}=1|Y_{ti1},\ldots ,Y_{ti\left(j-1\right)}\right)=\alpha_{tj}+\beta_{tj}Y_{ti\left(j-1\right)}\text{,}$$

for j=6,…,10 and t=1,…,7.

We analysed these data using monotone multiple imputation to impute the missing outcomes. Following Eq. (2), the imputation model for each outcome was a logistic regression on the previous outcome, treatment group and their interaction. We performed the imputation using SAS PROC MI and R. In R, the MICE software library (van Buuren and Oudshoorn, 2000) is not explicitly designed for monotone imputation, but was made to implement it by suitable specification of the imputation models and setting the number of cycles to 1. Programs are available as supplementary material in the electronic version of this paper. In anticipation of instability (due to the lack of variation in the outcomes and the high dropout rate), and in order to reduce Monte Carlo error, in each method we use a relatively large number of 100 imputations.

The results (Table 1) show that the point estimates (estimated log odds of outcome) and their standard errors vary substantially between the two MI methods, especially for $\delta_{7}$. The observed differences are not attributable to Monte Carlo error in the MI procedure, because Monte Carlo standard errors were no more than 0.08; these were computed by a jackknife method using the mim command in Stata (Royston et al., 2009). Substantial differences are also seen between the MI and CC analyses. These are not expected to agree precisely, because they make different assumptions about the missing data mechanism, but it is a cause for concern that the standard errors for some of the parameters are substantially larger for the MI analyses than for the CC analysis. Further, the fractions of missing information, which measure the increase in variance of the parameter estimates due to the missing data (Schafer, 1997), are unreasonably large, reaching 0.95 for $\delta_{7}$.

The sparseness of the data implies that some parameter estimates in model (2) are infinite. The differences between procedures may therefore be a consequence of different ways of handling perfect prediction. We return to these data in Section 6 where we explore to what extent handling of perfect prediction can explain the differences.

## 3 Theory

### 3.1 Imputation model

We assume that the data for individual i are $\left(X_{i},Y_{i}\right)$ where multivariate X is complete but univariate Y is incomplete. We focus on the case where Y is discrete. Our aim is to impute the missing values of Y: this may either be the whole imputation task, part of a monotone imputation procedure, or part of a MICE algorithm. Assuming individuals are independent, the imputation model is $p\left(y|x,\theta \right)=p\left(Y_{i}=y|X_{i}=x,\theta \right)$. If X is discrete then our model could be the saturated model $p\left(y|x,\theta \right)=\theta_{xy}$. More commonly, X includes discrete and continuous components, and modelling assumptions are required. We will consider the three models most commonly used in practice. When Y is binary, we consider the logistic model

(3) $$\mathrm{logit}\;p\left(Y_{i}=1|X_{i}=x,\theta \right)=\alpha +\beta^{\prime }x$$

where β and x are p×1 vectors. When Y is an ordered categorical variable with levels 1,…,k for k>2, we consider the ordered logistic model

(4) $$\log p\left(Y_{i}>y|X_{i}=x,\theta \right)=\alpha_{y}+\beta^{\prime }x\text{.}$$

When Y is a nominal categorical variable, we consider the multinomial model

(5) $$\log p\left(Y_{i}=y|X_{i}=x,\theta \right)=\alpha_{y}+\beta_{y}^{\prime }x$$

where $\alpha_{1}=\beta_{1}=0$.

### 3.2 Proper imputation

In order for Rubin’s rules to produce valid results, multiple imputation must allow for uncertainty in the parameters of the imputation model. We first consider three ways of doing this (Rubin and Schenker, 1986).

#### 3.2.1 Explicitly Bayesian methods

Much of the literature concerns the problem of imputing a binary (or other discrete) incomplete variable within strata defined by one or more other discrete variables (Rubin and Schenker, 1986). In this case, a prior such as Beta(1,1) may be used for the stratum-specific probability π of the incomplete variable. A draw $\pi^{*}$ from the posterior distribution of π is easily obtained and directly leads to a set of proper imputations. This method is applicable to the dental pain trial but does not extend easily to a more general regression situation.

#### 3.2.2 Bootstrap

Rubin and Schenker (1986) propose a method that they call the approximate Bayesian bootstrap (Rubin, 1981) to draw proper imputations in the discrete case. This may be generalised by drawing a bootstrap sample of the observed data and re-fitting the imputation model p(y|x;θ) to the bootstrap sample, yielding a parameter $\theta^{*}$ and hence imputations $y^{*}$ drawn from $p\left(y|x;\theta^{*}\right)$ (Royston, 2004, 2005).

#### 3.2.3 Normal-approximation draw

This method is widely used in multiple imputation software. Let the maximum likelihood estimate from fitting the imputation model p(y|x;θ) be $\overset{ˆ}{\theta }$ with variance-covariance matrix $\overset{ˆ}{V}$. If the log-likelihood is approximately quadratic and the prior is weak, then the posterior is approximately $\theta |y∼N\left(\overset{ˆ}{\theta },\overset{ˆ}{V}\right)$. We therefore draw $\theta^{*}$ from this distribution and obtain imputations $y^{*}$ from $p\left(y|x;\theta^{*}\right)$. For linear regression, this method is exact, provided it is modified by first drawing the variance from its exact inverse-gamma posterior.

### 3.3 Perfect prediction

Perfect prediction may occur in any GLM with a categorical outcome. In this case, the likelihood tends to a limit as one or more regression parameters go to plus or minus infinity: loosely, these parameters have maximum likelihood estimate (MLE) equal to plus or minus infinity. It is arguable whether this is in itself a problem, since odds ratios of 0 or infinity should be no more surprising than estimated probabilities of zero or one. However, a problem definitely arises with standard errors computed from the information matrix: these are extremely large, reflecting the near-flat nature of the likelihood.

Software packages differ in their handling of perfect prediction in usual modelling situations. SAS PROC LOGISTIC, for example, prints a warning message but does not modify its estimation procedure, so that extremely large standard errors are reported. Stata’s logistic procedure (StataCorp, 2009), by contrast, attempts to detect perfect prediction before fitting the model. If perfect prediction is detected, the perfectly predicting covariate and the perfectly predicted observations are dropped from the analysis. If perfect prediction is not detected, the singular information matrix is taken to imply a non-identifiable model, and standard errors are computed via a generalised inverse approach.

## 4 Proper imputation in the presence of perfect prediction

We now show how the Normal-approximation draw method of Section 3.2.3 fails if perfect prediction arises. We use an artificial data set comprising a complete binary variable X and an incomplete binary variable Y (Table 2). In this section we focus on imputing the missing values of Y when X=0. The relevant observed data are 100 failures and no successes, so under the MAR assumption, we would assume that the missing values are all (or almost all) failures.

**Fig. 1:**
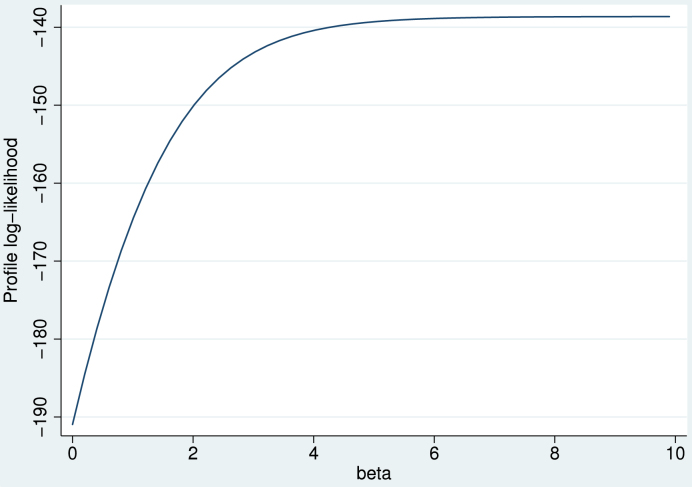
Profile log-likelihood for model logitp(Y=1|X=x)=α+βx fitted to the data in Table 2.

When we fit the logistic regression logitp(Y=1|X=x)=α+βx to the complete cases, there is no finite MLE (Fig. 1), reflecting perfect prediction. If no corrections are made, then the software will report a very large estimate of β and an even larger standard error. If this standard error is taken at face value and the Normal-approximation draw method is applied, then $\beta^{*}$ will be drawn from a very wide and flat posterior distribution, in such a way that most draws will be either very large and positive or very large and negative. In our data, the 100 missing observations will thus typically be imputed either correctly as 100 failures, or incorrectly as 100 successes. This will downwardly bias the point estimate of p(Y=1|X=0) and inflate its standard error by the large between-imputation variability. We call this method “Normal/allow”.

One alternative to “Normal/allow” arises if the software detects the large standard errors and adopts a generalised inverse approach. A large between-imputation variability is avoided, but the precise consequences depend on the generalised inverse method used. A second alternative arises if the software detects perfect prediction before model fitting and drops the perfectly predicting covariate and the perfectly predicted observations from the analysis. Then we are left with the simple model logitp(Y|X=1)=α for which the MLE is clearly $\overset{ˆ}{\alpha }=\log\left(100/100\right)=0$ (i.e. p(Y|X=1)=0.5). It would be an error to apply this model to observations with X=0.

## 5 Solutions

The perfect prediction problem can occur whenever a Normal-approximation draw method is used. The alternatives suggested in Section 3, the explicitly Bayesian method and the bootstrap, therefore avoid the perfect prediction problem. Here we propose two more solutions within the Normal-approximation draw method.

### 5.1 Penalised regression

Firth (1993) proposed a general penalised regression procedure which eliminates the first-order bias of the parameter estimates; more importantly for the present work, it also avoids infinite parameter estimates. For logistic regression, this procedure can be expressed as a modification of the iteratively reweighted least squares algorithm (McCullagh and Nelder, 1989). The ith observation is augmented with one success and one failure, both carrying weight $h_{i}/2$, where $h_{i}$ is the ith diagonal element of the weighted hat matrix $W^{1/2}X{\left(X^{T}WX\right)}^{-1}X^{T}W^{1/2},W$ is the diagonal matrix of true variances which depends on the parameters β, and X is the design matrix. Penalised regression gives more appropriate standard errors and more symmetrical log-likelihoods than unpenalised regression (Bull et al., 2007) so that a Normal-approximation draw is more appropriate than for “Normal/allow”. We call this method “Normal/penalise”. Depending on the implementation, it can be markedly slower than unpenalised regression, and hence computationally unrealistic in the MICE context where the regression must be performed repeatedly. Also, implementation for the ordered logistic and multinomial regression models is harder (Bull et al., 2002).

### 5.2 Regression with augmented data

We propose an *ad hoc* but computationally convenient way to avoid the problems associated with perfect prediction: we augment the data with a few extra observations that avert perfect prediction. This is done for each predictor variable by adding observations at two design points differing only on that predictor. The added observations are assigned a small weight to limit their impact on the estimated imputation model. This augmentation method has been implemented in our Stata software, ice (Royston, 2004, 2005).

Our algorithm is as follows. We first compute the mean ${\overset{¯}{x}}_{j}$ and SD $s_{j}$ of each predictor $X_{j}$. We then add 2 records to the data, where $X_{1}$ is either ${\overset{¯}{x}}_{1}-s_{1}$ or ${\overset{¯}{x}}_{1}+s_{1};Y$ takes its first observed level; and other variables $X_{j}\left(j\neq 1\right)$ are fixed to their mean ${\overset{¯}{x}}_{j}$. We then repeat this procedure for each level of Y and for each predictor variable $X_{j}$. If there are p predictors and Y has k levels then this adds a total of 2pk records to the data. The same method is used whether the predictor is quantitative or a dummy for a categorical variable; it is therefore not exactly invariant to the choice of reference category for categorical variables with more than 2 levels.

We now assign a small weight w to each of these added observations. We propose w=(p+1)/2pk so that the total added weight is W=p+1, the total number of parameters in the imputation model. This agrees with the method of Firth (1993) which eliminates first-order bias in parameter estimates, with the method of Clogg et al. (1991), and with standard practice in the 2-by-2 table where 0.5 is added to all cells if any cell is zero (Cox and Snell, 1989; Sweeting et al., 2004). Again, a Normal-approximation draw is more appropriate than for “Normal/allow”. We call this method “Normal/augment”.

**Fig. 2:**
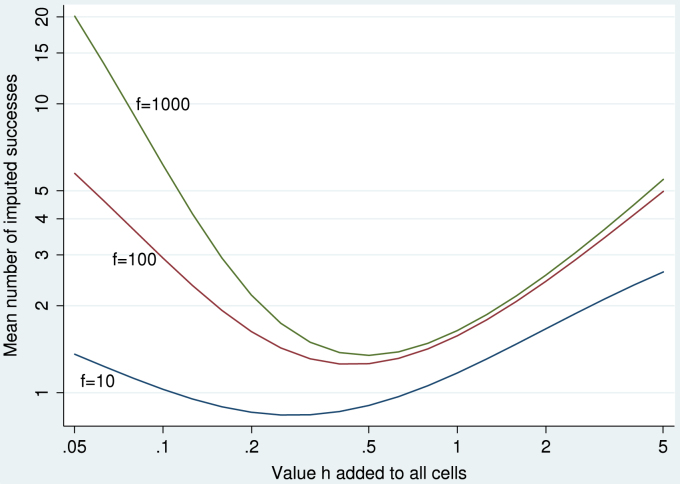
Mean number of imputed successes in data in Table 2 using “Normal/augment” method: comparison of different values h added to all cells, for three different numbers of failures f.

To support the above choice of w, we performed a small comparison of assigning different weights to the added observations. We focus on a single group with r=0 successes and f failures. The X=0 group in Table 2 has r=0 and f=100, and we also explored the cases f=10 and f=1000. When the Normal approximation draw method is used with h added to all cells, the mean number of imputed successes is E[exp(m+Zs)/{1+exp(m+Zs)}] where Z∼N(0,1), m=log{(r+h)/(f+h)} and $s=\sqrt{1/\left(r+h\right)+1/\left(f+h\right)}$. This expectation was computed using Gauss–Hermite quadrature; a low value is desirable. Fig. 2 shows that the mean number of imputed successes is near 1 if h≈0.5, with only weak dependence on f, but it increases as h moves away from 0.5. By comparison, the Bayesian approach with Beta(1,1) prior would impute a mean number of successes of f/(f+1) or just under 1. Thus $h\approx \frac{1}{2}$, which corresponds to W≈p+1, appears to be a good choice.

### 5.3 Method comparison

**Fig. 3:**
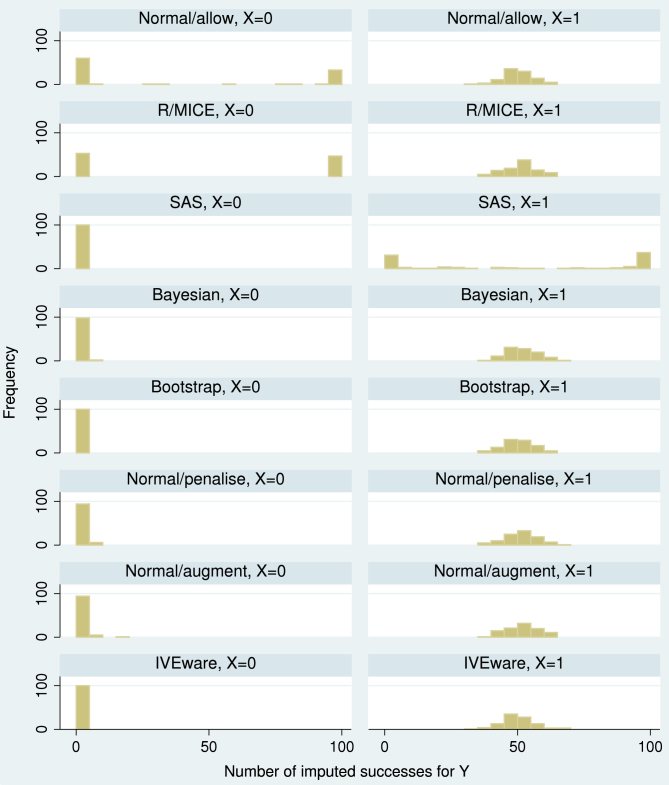
Artificial data of Table 2: number of imputed successes in 100 individuals with X=0 (left panels) and in 100 individuals with X=1 (right panels), using various analysis methods and software packages.

Fig. 3 shows the distribution of the number of imputed successes in each of 100 imputed data sets for the data in Table 2. Recall that imputed successes should be very few in the X=0 group and about half of the X=1 group.

The first three rows of Fig. 3 show methods that do not work satisfactorily. The Normal/allow method performs very badly in the group with perfect prediction (X=0). Imputation with R/MICE appears to be using the Normal/allow method. The code used was

**Figure:**
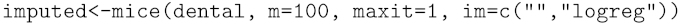

Surprisingly, imputation with SAS gives over-variable imputations in the group *without* perfect prediction (X=1). These results presumably result from an unsuccessful attempt to correct the singular variance-covariance matrix. The SAS code was:

**Figure:**
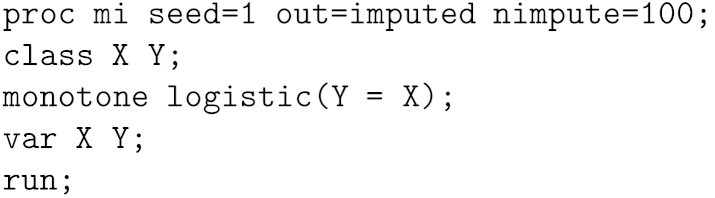

The next four rows of Fig. 3 show the methods proposed, which all perform satisfactorily. They differ slightly in that the bootstrap method imputes no successes in X=0 whereas the other methods impute a small number of successes in this group. We return to this point in the discussion. The Normal/augment method was implemented in Stata using

**Figure:**
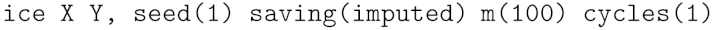

The last row of Fig. 3 shows results using IVEware in SAS (Raghunathan et al., 2001, 2007). This performed well for these data, but it appeared to achieve this by replacing very large standard errors with zero.

### 5.4 More than two levels

We also considered the case of an incomplete variable with 3 levels, by adding to Table 2 100 observations with X=0 and Y=2, and 100 observations with X=1 and Y=2. This leads to perfect prediction when Y is imputed using the multinomial model (5). The average fraction of the 100 observations with X=0 and missing Y that were imputed as 1 was 42% using Normal/allow, 0.7% using Normal/augment, and 0% using the bootstrap.

## 6 Revisiting the dental pain data

**Fig. 4:**
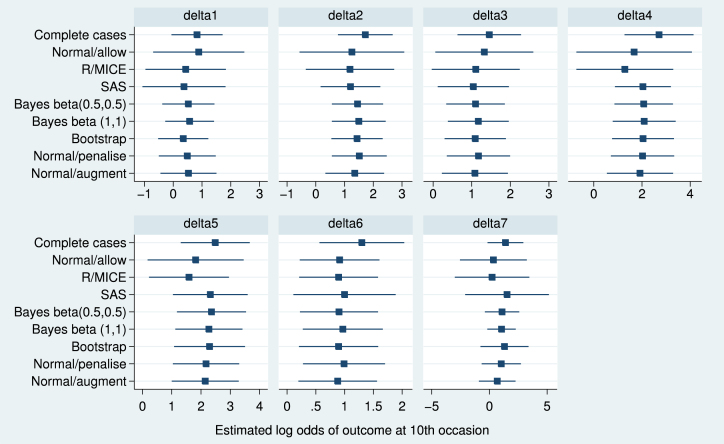
Dental pain data: estimated log odds of pain relief at 10th occasion (with 95% confidence interval) for various analysis methods.

We now return to the dental pain data. Further exploration of the data shows that perfect prediction occurred at some point in every treatment group except group 6. This suggests that the results from SAS and R/MICE, seen in Table 1, are likely to be wrong. We compare those results with results using the explicitly Bayesian, penalised, bootstrap and augmentation methods (Fig. 4). The Bayesian method was done both with beta (0.5, 0.5) and beta (1, 1) priors. The augmentation method was implemented in Stata using code available as supplementary material in the electronic version of this paper.

It is clear from Fig. 4 that Normal/allow, R/MICE and SAS can give seriously misleading results. The newly introduced methods are reasonably consistent with one another, although differences are noticeable even between the Bayesian methods with different priors, presumably because of the extreme sparseness of the data. Estimated fractions of missing information with the newly introduced methods ranged from 0.73 to 0.85 for $\delta_{7}$ and were lower for the other parameters.

The complete cases analysis gives larger point estimates than the newly introduced methods. The difference is not unexpected, since complete cases analysis makes a different assumption from MI analysis. In these data, missing data almost always occur when there was no pain relief at the previous outcome, so complete cases analysis tends to exclude individuals with worse outcomes: this explains the differences seen.

## 7 Simulation study

We finally perform a small simulation study to explore the properties of the proposed methods in a more realistic setting with three covariates (two binary and one continuous) and a continuous outcome. 1000 data sets were simulated, each with a sample size n=500. $X_{1}$ was binary with $p\left(X_{1}=1\right)=\pi_{1}$. $X_{2}$ was binary with $p\left(X_{2}=1|X_{1}=0\right)=\pi_{2}$ and $p\left(X_{2}=1|X_{1}=1\right)=0$ so that perfect prediction always occurred. $X_{3}$ was continuous with $X_{3}|X_{1},X_{2}∼N\left(0,1\right)$. Y was continuous with $Y|X_{1},X_{2},X_{3}∼N\left(\beta_{1}X_{1}+\beta_{2}X_{2}+\beta_{3}X_{3},\sigma^{2}\right)$. $X_{1}$ was missing completely at random with probability α; the other variables were complete. We fixed $\pi_{1}=0.1,\pi_{2}=0.8,\beta_{1}=\beta_{2}=1$, $\beta_{3}=0.5,\sigma =2$ and α=0.3. The missing values of $X_{1}$ were imputed from a logistic regression on $X_{2},X_{3}$ and Y, and perfect prediction was handled by the Normal/allow, Normal/augment, Normal/penalise and Bootstrap methods. Five imputed data sets were created, although more would be needed for definitive results in any particular data set (Bodner, 2008). The imputed data were used to estimate $\pi_{1},\beta_{1},\beta_{2}$ and $\beta_{3}$.

The results (Table 3) confirm the poor performance of Normal/allow for all parameters except $\beta_{3}$: the estimated prevalence of $X_{1}$ is biased upwards and estimated associations are weakened. Coverages for Normal/allow are reasonable, but only because bias is accompanied by inflated standard errors. The other three methods perform equally well, with negligible bias, similar empirical standard errors, appropriate model-based standard errors, appropriate coverage and similar power.

## 8 Discussion

We have shown that implementations of imputation procedures for categorical variables can go seriously wrong if perfect prediction occurs. This was a problem in earlier versions of our own software, ice, and at the time of writing it remains a problem in at least two other major software packages. It can apply in any setting where an imputation procedure involves a categorical data regression and where the uncertainty about the imputation model parameters is allowed for by sampling from a Normal approximation to a posterior distribution.

General advice is that imputation models should aim to include as many relevant variables as possible in order to make the missing at random assumption more plausible (Collins et al., 2001; Zhou et al., 2001). While we agree with this argument, it does increase the probability of perfect prediction occurring. Further research is needed to be sure that there are no other hidden problems that can arise with large imputation models.

Various approaches to perfect prediction are possible. The simplest is to abandon proper imputation if perfect prediction is detected — that is, to use the estimated parameter $\overset{ˆ}{\theta }$ rather than a draw $\theta^{*}$ from the posterior. This certainly avoids the traps we have described, but it somewhat underestimates the uncertainty in the data if Rubin’s rules are applied. We have proposed three general alternatives: drawing approximately from the posterior via a bootstrap procedure, using penalised regression, and augmentation. A fourth alternative, the explicitly Bayesian method, appears to be used by the new mi package for R (Gelman et al., 2008), which uses a default “minimal-information” prior distribution via Gelman’s bayesglm function. The bootstrap procedure and augmentation are probably the most straightforward of these alternatives.

Perfect prediction may be regarded as logical (“X=0 always implies Y≠1”) or as random (“X=0 can occur with Y=1, but this was not observed in our data”). The bootstrap procedure implicitly views perfect prediction as a logical phenomenon, whereas the augmentation procedure implicitly views it as random (the X=0 group in Fig. 3 has a few successes imputed by Normal/augment, but none by Bootstrap). This distinction could help in choosing which method to use.

One way to detect problems with a Normal approximation to the log likelihood logl(θ) is to compare the values of $l\left(\theta^{*}\right)/l\left(\overset{ˆ}{\theta }\right)$ from the true and approximated likelihoods (Raghunathan et al., 2001). The ratio R of the two values should be close to 1 but can be very large (more than 1000) when perfect prediction is inappropriately handled. We suggest that values outside the range (0.1,10) should be a cause for concern. One way to improve the approximation is to use importance sampling in which several values of $\theta^{*}$ are drawn from the Normal approximation, and the values of R are computed and used in choosing one of the $\theta^{*}$ values (Rubin, 1987b, 2003).

We have implemented the augmentation method in version 1.4.1 and later of the ice command for Stata. Users should be aware that (unlike in methods such as penalised likelihood) different parameterisations–for example, different choices of reference category for a categorical variable–can give somewhat different answers. However, in our experience, such differences are of no practical importance, and the main problem that can arise is upward bias in the prevalence of rare levels of categorical variables. Prevalences will be biased when the number of augmented events in a level (approximately (p+1)/k) is not small compared with the number of observed events.

In summary, awareness of the perfect prediction problem is important for users of MI routines, since these may not always handle the problem appropriately. It is also essential for anyone programming an MI routine.

## Figures and Tables

**Table 1:** Dental pain trial: comparison of complete cases and 2 different MI procedures for the logistic regression of outcome on treatment group at the final timepoint. Figures are estimated log odds of pain relief (standard error) and fraction of missing information (FMI) in model (1).

Parameter	MI with 100 imputations (n=366)				Complete cases (n=216)
	SAS/PROC MI		R/MICE		
	Estimate (s.e.)	FMI	Estimate (s.e.)	FMI	
$\delta_{1}$	0.37 (0.74)	0.84	0.43 (0.71)	0.83	0.83 (0.45)
$\delta_{2}$	1.21 (0.53)	0.59	1.19 (0.78)	0.80	1.72 (0.49)
$\delta_{3}$	1.04 (0.47)	0.53	1.10 (0.58)	0.67	1.46 (0.42)
$\delta_{4}$	2.04 (0.59)	0.44	1.29 (1.02)	0.85	2.71 (0.73)
$\delta_{5}$	2.32 (0.65)	0.41	1.59 (0.70)	0.69	2.48 (0.60)
$\delta_{6}$	1.00 (0.45)	0.51	0.90 (0.35)	0.23	1.30 (0.38)
$\delta_{7}$	1.52 (1.85)	0.92	0.23 (1.65)	0.95	1.39 (0.79)

**Table 2:** Artificial data used to illustrate the perfect prediction problem.

X	Y		
	0	1	Missing
0	100	0	100
1	100	100	100

**Table 3:** Results of simulation study to compare Normal/allow, Bootstrap, Normal/penalise and Normal/augment methods for handling perfect prediction. EmpSE is the empirical standard error, ModSE is the relative error in the model standard error compared with the empirical standard error, Coverage is coverage of a nominal 95% confidence interval, and Power is the power to reject parameter = 0.

Parameter	Method	Bias	EmpSE	ModSE (%)	Coverage (%)	Power (%)
$\pi_{1}$ = 0.1	Normal/allow	0.07	0.05	130	99	23
	Bootstrap	0.00	0.02	1	95	100
	Normal/penalise	0.00	0.02	3	95	100
	Normal/augment	0.00	0.02	3	95	100
$\beta_{1}$ = 1	Normal/allow	−0.22	0.36	47	93	20
	Bootstrap	0.02	0.43	1	94	65
	Normal/penalise	0.00	0.42	2	94	65
	Normal/augment	−0.02	0.42	3	95	63
$\beta_{2}$ = 1	Normal/allow	−0.11	0.24	26	96	90
	Bootstrap	0.01	0.25	1	96	98
	Normal/penalise	0.00	0.25	1	95	97
	Normal/augment	0.00	0.25	2	96	97
$\beta_{3}$ = 0.5	Normal/allow	0.00	0.09	−3	94	100
	Bootstrap	0.00	0.09	−3	94	100
	Normal/penalise	0.00	0.09	−3	94	100
	Normal/augment	0.00	0.09	−3	94	100
Maximum Monte Carlo error		0.01	0.01	5	1	2

## Appendix Supplementary data

MMC 1 Supplementary data for “Avoiding bias due to perfect prediction in multiple imputation of incomplete categorical variables”.
